# Catamenial pneumothorax: Not only VATS diagnosis

**DOI:** 10.3389/fsurg.2023.1156465

**Published:** 2023-04-04

**Authors:** Rosatea Quercia, Angela De Palma, Francesco De Blasi, Graziana Carleo, Giulia De Iaco, Teodora Panza, Giuseppe Garofalo, Valentina Simone, Michele Costantino, Giuseppe Marulli

**Affiliations:** Unit of Thoracic Surgery, Department of Precision and Regenerative Medicine and Ionian Area, University of Bari “Aldo Moro”, Bari, Italy

**Keywords:** catamenial pneumothorax, thoracic endometriosis, VATS, diagnosis, surgical treatment

## Abstract

**Background:**

Catamenial pneumothorax (CP) is a rare type of spontaneous, recurring pneumothorax occurring in women, from the day before menstruation until 72 hours after its beginning. Conservative treatment is generally associated with recurrence of CP. Video-assisted thoracic surgery (VATS) approach allows not only to obtain diagnosis but also to guide definitive treatment of causing lesions, such as ectopic endometrial implants or diaphragmatic defects and fenestrations. We report our experience in VATS management of CP to focus on its role in CP.

**Materials and methods:**

In this retrospective observational study, we collected data from women referred to our center for CP, from January 2019 to April 2022. All patients underwent VATS approach, with muscle-sparing thoracotomy when diaphragmatic fenestrations were detected, to perform selective diaphragmatic plication and/or partial diaphragmatic resection. Results were analyzed in terms of pneumothorax recurrence after surgical treatment. All patients were referred to gynecologists for medical therapy.

**Results:**

Eight women (median age 36 years, range: 21–45), all with right side CP, were included; three already had pelvic endometriosis and two had already undergone lung apicectomy at other institutions. VATS allowed us to detect diaphragmatic fenestrations in seven patients (87.5%) and apical bullae in five (62.5%). Apicectomy was performed in five cases (62.5%), selective diaphragmatic plication in two (25%), and partial diaphragmatic resection in five (62.5%). Chemical pleurodesis with talc was performed in all to minimize the risk of recurrence. Pathological diagnosis of endometriosis on the resected diaphragm was achieved in five patients (62.5%). No recurrence occurred, except for one woman who stopped medical treatment for endometriosis.

**Conclusions:**

In the management of patients with CP, VATS should be recommended not only to obtain an explorative diagnosis of ectopic endometrial implants or diaphragmatic fenestrations but also to allow the most appropriate surgical treatment and obtain pathological specimens for confirmation and definitive diagnosis of thoracic endometriosis. Medical therapy to achieve ovarian rest is mandatory in the postoperative period and should not be discontinued.

## 1. Introduction

Endometriosis is a common, benign condition characterized by the localization of endometrial-like glands and stroma outside the uterine cavity. The thoracic cavity is the most common site of endometriosis outside of the abdominal-pelvic cavity, where it can produce a range of clinical and radiological manifestations including catamenial pneumothorax (CP), catamenial hemothorax, catamenial hemoptysis, and pulmonary nodules, also known as thoracic endometriosis syndrome (TES) (1–3).

CP is the most common manifestation (73%) of TES and is a rare type of spontaneous, recurring pneumothorax occurring in women of reproductive age (4), from 24 hours before menstruations until 72 hours after their beginning (5), but the literature indicates also other time criteria, up to 7 days before and after monthly bleeding (6). CP could be characterized by the presence or not of thoracic endometriosis (7, 8) (Table 1).

**Table 1:** Classification of recurrent spontaneous pneumothoraces in women of reproductive age referred for surgical treatment, in the absence of a known underlying disease [modified from Visouli et al. (8)].

Definition	Criteria	Percentage	Total percentage
Catamenial/endometriosis-related	Recurrent, in temporal relationship with menses with evidence of thoracic endometriosis	15.4	23.7
Catamenial/non-endometriosis-related	Recurrent, in temporal relationship with menses without evidence of thoracic endometriosis	8.3	
Non-catamenial/endometriosis-related	Occurring in the intermenstrual period with evidence of thoracic endometriosis	7.7	76.3
Non-catamenial/non-endometriosis-related	Occurring in the intermenstrual period without evidence of thoracic endometriosis	68.6	

Differently from primary spontaneous pneumothorax (9), the mean age of women with CP is 34–37 years (6, 7, 10, 11). The experienced symptoms are comparable to those of spontaneous pneumothorax and consist of pleuritic chest pain, cough, and shortness of breath (9). Diaphragmatic irritation may produce referred pain to the periscapular region or radiation to the neck (most often right-sided). In most cases (95%), there is involvement of the right hemithorax, in 5% of cases of the left hemithorax, and in 3% bilateral involvement (11).

Chest x-ray is the first imaging exam for the diagnosis of pneumothorax. CP may be associated with thoracic endometriosis or diaphragmatic fenestrations (6, 12), and computed tomography (CT) or magnetic resonance imaging (MRI) could show small diaphragmatic defects, called “air-filled bubble” perforations (13). In particular cases, when patients suffer from abdominopelvic symptoms, abdominal MRI may be helpful in the diagnosis of endometriosis and subsequently of TES.

The gold standard diagnostic tool and treatment for CP is video-assisted thoracic surgery (VATS) (11, 14–17), which allows multiple treatment modalities depending upon the characteristics of identified lesions. In cases of superficial endometriotic implants, lesions could be fulgurated using bipolar diathermy, CO^2^ laser, Nd–YAG laser, argon laser, or plasma energy, while deeper endometriotic implants should be excised using sharp dissection (15, 16, 18–20), lung wedge resection with a stapling device, segmentectomy, or in rare cases lobectomy (14, 16, 21, 22). Pleurodesis, which can be accomplished chemically with talc or mechanically with pleural abrasion and partial pleurectomy, decreases the recurrence rate of CP after VATS by 20%–25% (16, 23–26). Proper CP diagnosis, especially if done with histological confirmation of the endometrial foci in the pleura, pulmonary parenchyma, or diaphragm, may be crucial to the patient even after surgical treatment of pneumothorax, because hormonal therapy may contribute to the avoidance of CP recurrence (27, 28).

We report our experience in VATS management of CP to focus on the role of VATS not only in obtaining the definitive diagnosis of endometriosis but also in selecting the most appropriate surgical treatment.

## 2. Materials and methods

In this retrospective observational study, we collected data from women of reproductive age referred to our center for recurrent spontaneous pneumothorax, from January 2019 to April 2022, selecting those having temporal relation with menses, compatible with CP.

Data were collected about age at first pneumothorax and at recurrence, onset symptoms, side of CP, history of smoke, time relationship with menses, medical history of endometriosis, imaging used for diagnosis, number of episodes of pneumothorax before surgical treatment, type and time of surgical treatment, results of VATS approach, complications after surgery, and hormonal therapy after surgery.

All patients underwent VATS approach, with muscle-sparing thoracotomy when diaphragmatic fenestrations were detected, to perform selective diaphragmatic plication and/or partial diaphragmatic resection.

Results were analyzed in terms of pneumothorax recurrence after surgical treatment.

All patients were referred to gynecologists for medical therapy. A telephone questionnaire was also submitted, regarding the gynecological therapeutic follow-up.

Continuous variables are reported as medians with range and categorical variables as counts and percentages.

## 3. Results

Twenty-two women of reproductive age were referred to our center for recurrent spontaneous pneumothorax. Eight (36.4%) of them had temporal relation with menses, compatible with CP.

The group of eight women had a median age of 37 years (range: 21–45) at the onset of symptoms, that is at the time of first pneumothorax episode, and the same at the time of recurrence, when they were operated on. No difference in the age of CP presentation was found between patients with and those without pelvic endometriosis. In one case, there was SARS-CoV-2 infection concurrent with the first episode of pneumothorax, but the recurrence occurred a month later.

Onset symptom was chest pain in all cases, associated with dyspnea in three. In all cases, the pneumothorax was on the right side. Two patients were smokers, while the remaining six consisted equally of three non-smokers and three ex-smokers.

In one patient, the pneumothorax occurred 1 day before the start of the menses, while in the other cases 48 hours after the start of the menses.

Three patients already had a diagnosis of pelvic endometriosis and all of them had already undergone abdominopelvic surgery. Estrogen–progestin therapy had been taken by these three patients (37.5% of the sample) for diagnosis of pelvic endometriosis, but this therapy was not for ovarian rest.

Half of the cases were women with a history of pregnancies with a median number of 2 (range: 1–2) deliveries.

**Figure 1:**
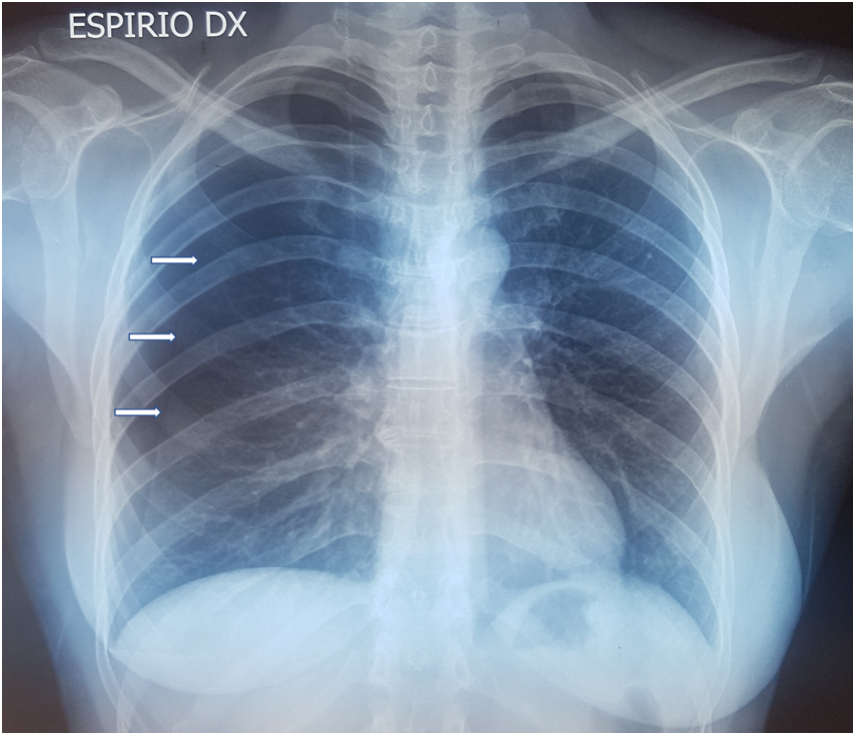
Chest x-ray showing CP on the right side: white arrows indicate the collapsed lung. CP, catamenial pneumothorax.

**Figure 2:**
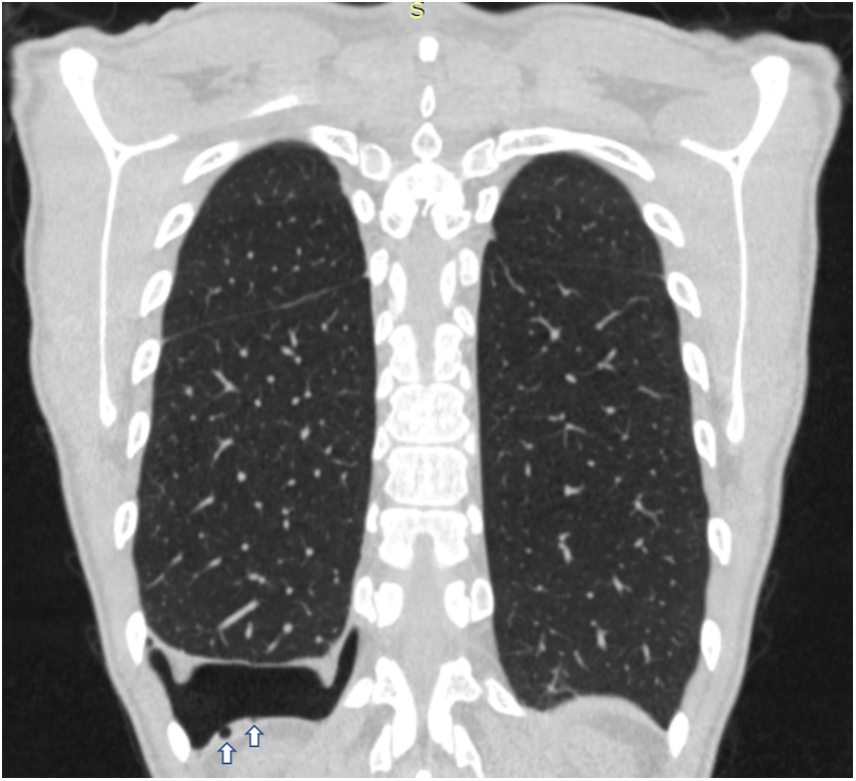
Coronal chest CT scan of a patient with CP showing on the right side small diaphragmatic defects, called “air-filled bubble” perforations (indicated by white arrows). CT, computed tomography; CP, catamenial pneumothorax.

In all patients, standard two-view chest x-ray was performed as first imaging exam for diagnosis (Figure 1). In three cases, preoperative chest CT was done, too: in one case, with recent SARS-CoV-2 infection, to exclude pneumonia or thromboembolic complications and in two cases because the patients had previously undergone surgery for recurrent pneumothorax at other institutions, without evidence of thoracic endometriosis. In the case with recent SARS-CoV-2 infection, CT was extremely helpful, revealing small diaphragmatic defects (“air-filled bubble” perforations) (Figure 2).

The median number of episodes of pneumothorax before surgical treatment was 3 (range: 2–4).

All patients underwent VATS surgical treatment. The median time to surgery, calculated as the difference between the date of admission and the date of surgery, was 2 days (range: 0–6 days).

**Figure 3:**
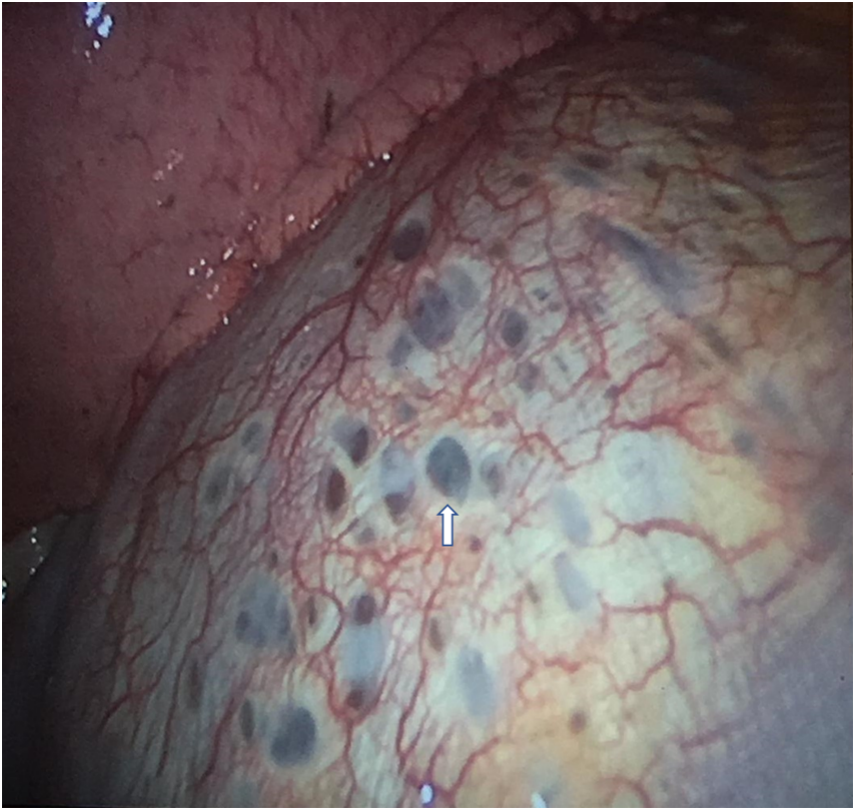
VATS intraoperative finding of typical diaphragmatic fenestrations (one indicated by white arrow), located at the central tendon of the diaphragm, in a patient with CP thoracic endometriosis-related. VATS, video-assisted thoracic surgery; CP, catamenial pneumothorax.

**Figure 4:**
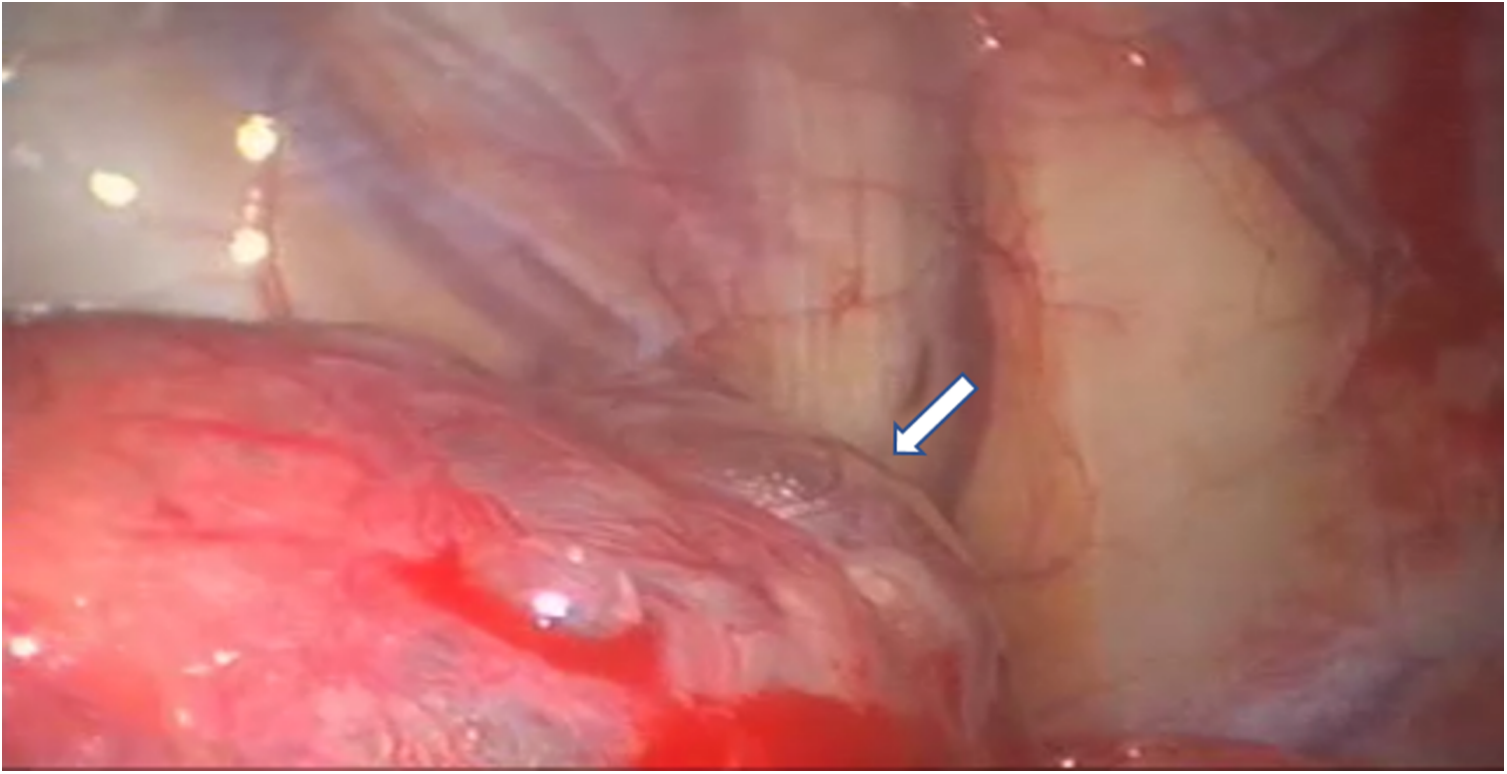
VATS intraoperative finding of dystrophic lung apex, characterized by the presence of apical blebs (indicated by white arrows), in a patient with CP non-thoracic endometriosis-related. VATS, video-assisted thoracic surgery; CP, catamenial pneumothorax.

VATS approach allowed to diagnose seven cases (87.5%) of CP thoracic endometriosis-related, with diaphragmatic fenestrations (Figure 3), associated with lung apical blebs and/or bullae in four of them and one case (12.5%) of CP non-thoracic endometriosis-related, with dystrophic apex only (Figure 4).

**Figure 5:**
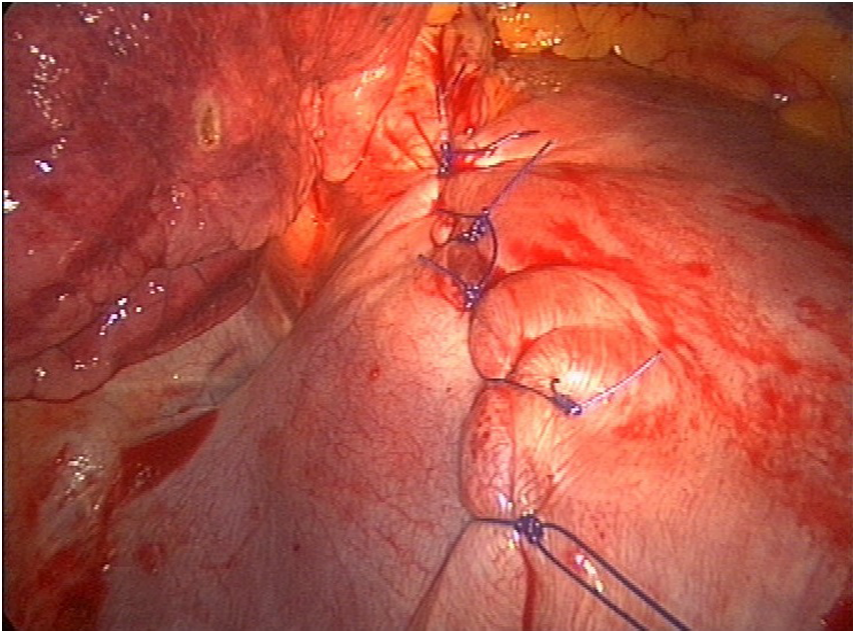
Selective diaphragmatic plication for VATS intraoperative finding of diaphragmatic fenestrations. VATS, video-assisted thoracic surgery.

**Figure 6:**
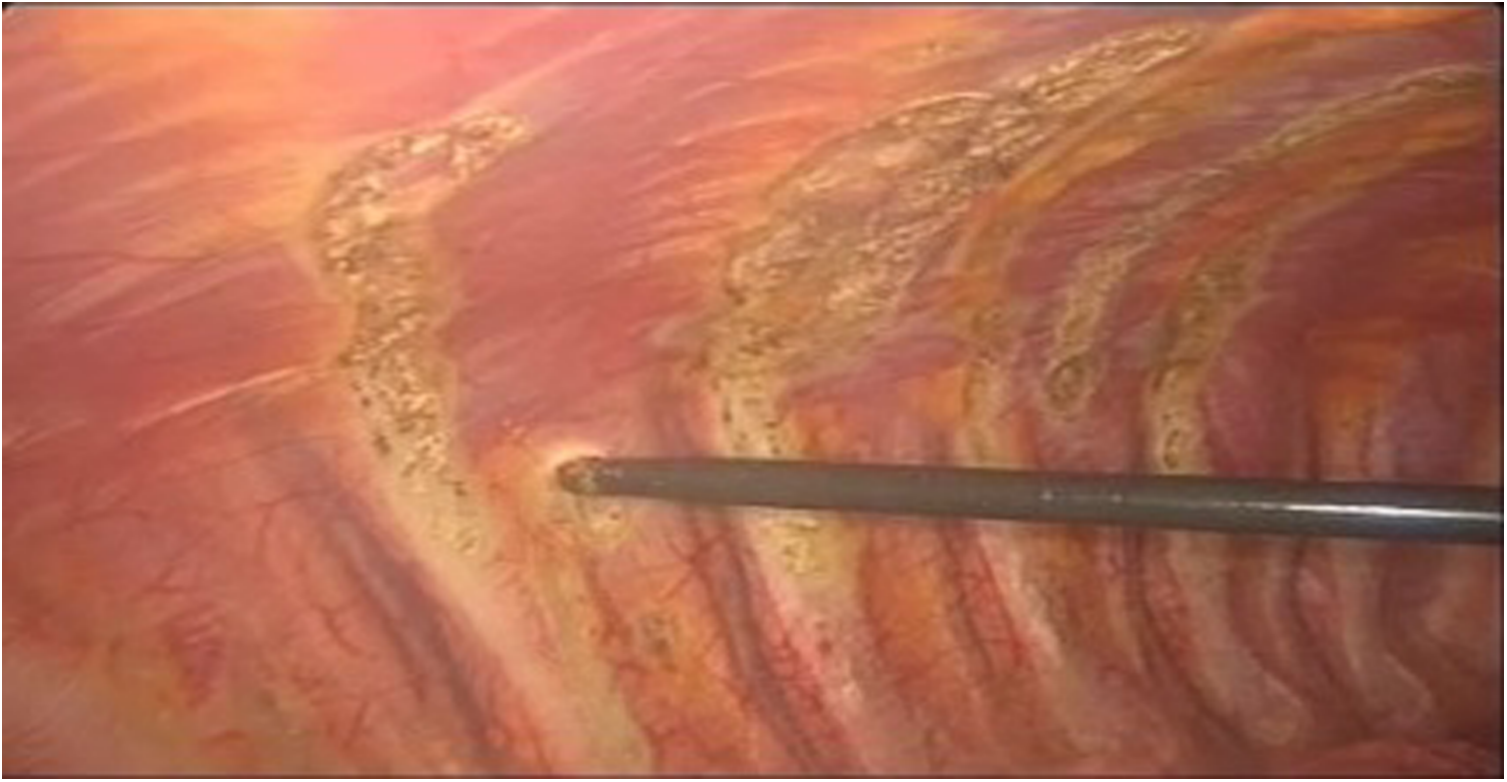
VATS mechanical pleurodesis with electrocautery on the parietal pleura to minimize the risk of CP recurrence. VATS, video-assisted thoracic surgery; CP, catamenial pneumothorax.

**Figure 7:**
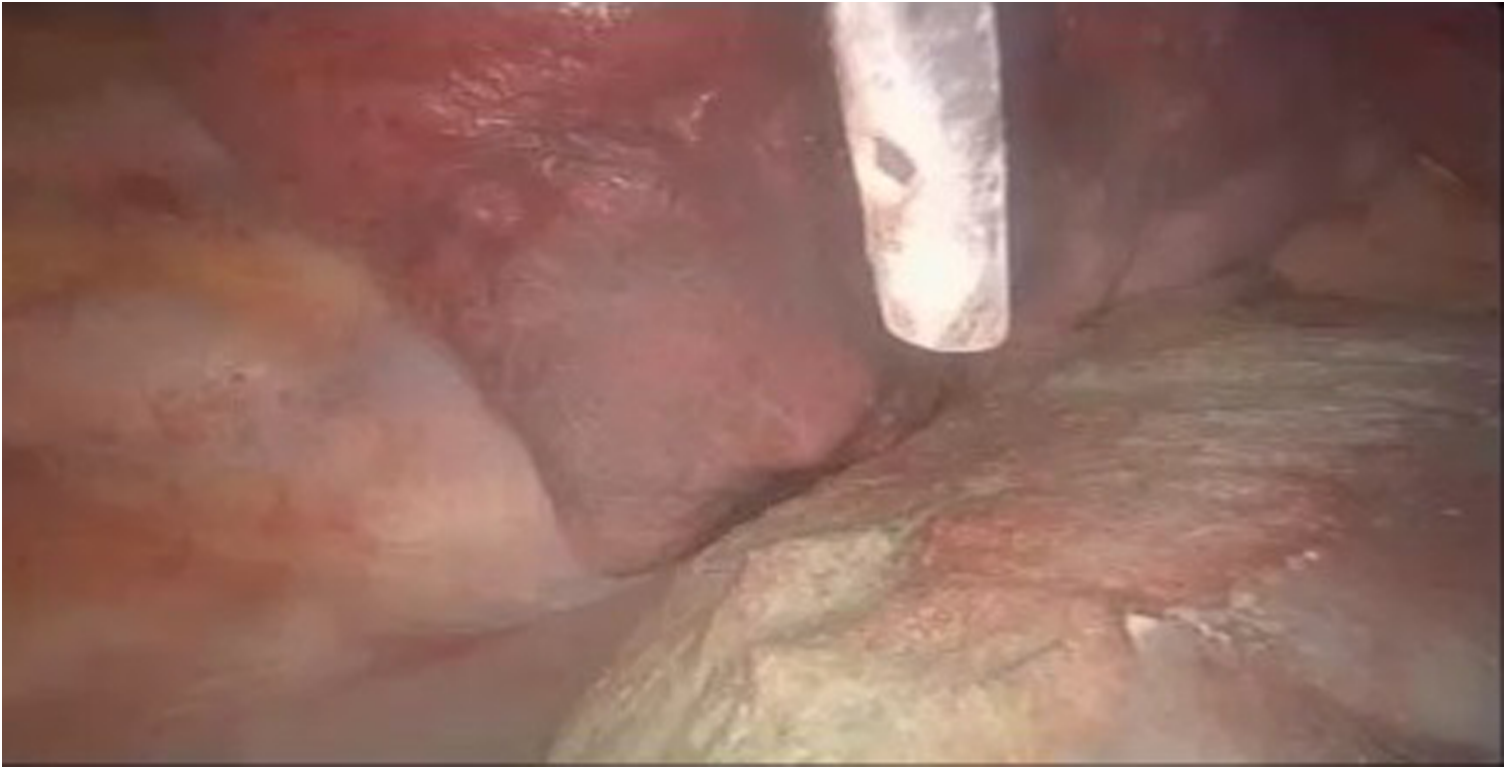
VATS chemical pleurodesis, nebulizing sterile medical talc powder on the pleura, to minimize the risk of CP recurrence. VATS, video-assisted thoracic surgery; CP, catamenial pneumothorax.

Because of the detection of diaphragmatic fenestrations, through a muscle-sparing thoracotomy, selective diaphragmatic plication was performed in two cases (25%) and partial diaphragmatic resection in five (62.5%) cases, with apposition of a prosthetic mesh in one of them (Figure 5). Lung apicectomy was performed in five cases (62.5%) for the evidence of dystrophic apex with blebs and/or bullae. In order to minimize the risk of recurrence, in all cases not only mechanical pleurodesis with electrocautery and/or brossage was performed, but also chemical pleurodesis, nebulizing sterile medical talc powder (Figures 6, 7).

No postoperative complications occurred. Pathological diagnosis of endometriosis on the resected diaphragm was achieved in five patients (62.5%).

All patients started hormonal therapy with estrogen–progestins after surgical treatment of pneumothorax, to be continued for at least 12–18 months.

The median follow-up period was 26 months (range: 7–55 months).

No recurrence of pneumothorax occurred after surgical treatment, except for one woman, approximately 32 months (970 days) later, who stopped medical treatment for endometriosis; this recurrence was simply and successfully treated with bed rest.

In two cases, medical therapy for endometriosis was discontinued, on average 3.5 years after thoracic surgery, due to unresponsiveness of pelvic endometriosis to treatment; thus, both women underwent hysteroannessiectomy. Another patient, after gynecological consultation, discontinued therapy 6 months after initiation, without presenting recurrence at 16 months.

## 4. Discussion

In our experience, at the onset of symptoms, the median age of patients with the first episode of CP was 37 years, similar to the literature (reported mean: 36.5 ± 6.8 years), and the median age at recurrence was the same (28).

No correlation was found with cigarette smoking or with/without previous deliveries.

All CP presented as unilateral and right-sided, in agreement with the literature (28).

Two-view chest x-ray was always performed in our patients, less often chest CT, and rarely abdominal MRI to look for pelvic endometriosis. There are no specific imaging diagnostic criteria, but chest CT sometimes can be helpful revealing small diaphragmatic defects called “air-filled bubbles” (13).

Diagnosis of CP is made mainly on the medical history (synchronicity with menses), while the diagnosis of thoracic endometriosis-related pneumothorax is based on intraoperative visual inspection and appropriate histological examination of the characteristic lesions.

The most frequent pathological findings reported in the literature were endometrial implants, present in 59.3% of patients, followed by diaphragmatic fenestrations in 57% and blebs/bullae in 25% (28). In our experience instead, seven patients (87.5%) had multiple diaphragmatic defects, usually located at the central tendon of the diaphragm, often adjacent to coexisting nodules and in four cases (50%) associated with dystrophic apex; in a patient, we found apical bullae only. Characteristic findings such as endometrial implants or diaphragmatic fenestrations may be absent in cases of CP, and blebs/bullae may be the only pathological findings, as in our last patient, while in some cases, there is no identifiable thoracic pathological abnormality (8).

Disease awareness (the size and the number of the characteristic lesions) with correct VATS timing in relationship with the menstrual cycle and meticulous inspection of the thorax, including the diaphragm, are important factors that need to be considered.

VATS approach is considered the treatment of choice, as it allows a better visualization of the endometriotic lesions, resection of all visible lesions and pleurodesis, and provides samples for pathological examination (11, 14–17). Moreover, VATS achieves better treatment results, mainly in term of less recurrences, in comparison to medical treatment alone (11, 14–17).

When extensive diaphragmatic repair is required, a video-assisted mini-thoracotomy or a muscle-sparing thoracotomy may offer better access to the diaphragm (8).

In our patients, the median time to surgery, calculated as the difference between the date of admission and the date of surgery, was 2 days. Such a short period from admission to intervention turns out to be essential to recognize endometriotic lesions which, by their own characteristics, are evident in the menstrual period. Thus, in our experience, in all patients, surgery was performed almost concurrently with the menses.

Histologic examination on samples collected during surgery was performed in 5/7 (71.4%) of our patients with CP thoracic endometriosis-related, with diaphragmatic lesions, and in all cases we obtained pathological diagnosis of thoracic endometriosis; in the remaining 2/7 patients (28.6%), only diaphragmatic plication was performed, without removal of tissues for histologic examination. Histologic examination was performed also in cases in whom diaphragmatic lesions were associated with intraoperative finding of blebs and/or bullae, and in the case with dystrophic apex only, but no endometriotic tissue nearby or within the bleb(s) and/or bulla(e) was found. Because of this last finding, we were able to diagnose a case of CP non-thoracic endometriosis-related.

In agreement with the literature, as we did in most of our patients (62.5%), in order to avoid recurrences, diaphragmatic resection with removal of endometrial implants is preferable to diaphragmatic plication; moreover, diaphragmatic coverage with a polyglactin or polypropylene mesh, a polytetrafluoroethylene (PTFE) mesh, or a bovine pericardial patch have been reported with good mid-term results (8). In our experience, the criteria used for performing a plication or a partial resection of the diaphragm were dependent on the extent of the involvement of the diaphragm: in case of diaphragmatic fenestrations involving an isolated area ≤5 cm^2^ of the diaphragm, we preferred to perform a partial resection, while in case of diaphragmatic fenestrations involving an area >5 cm^2^ or multiple diaphragmatic fenestration at different sites (potentially requiring multiple resection of the diaphragm), we decided to perform a plication.

All patients underwent ovarian rest therapy after surgical treatment of pneumothorax. Medical treatment of endometriosis utilizes gonadotropin-releasing hormone (GnRH) analogs, which block the ovarian hormones leading to amenorrhea (8); low doses of such drugs should be combined with female hormones (i.e., low-dose progestins) to reduce climacteric-like symptoms and improve tolerability and adherence to therapy (29). Most authors suggest administering this therapy in the immediate postoperative period, for 6–12 months, in all patients with proven catamenial and/or endometriosis-related pneumothorax (8). Low-dose oral contraceptives (estrogen–progestin) can also be used to treat endometriosis, but the literature data are conflicting (29). However, these treatments do not eradicate the disease. Moreover, one-third of the women with endometriosis do not respond to estrogen–progestins, which may be in part due to progesterone resistance (29, 30).

A period of exposure to hormonal therapy of at least 18 months along with surgical treatment was found to be essential to avoid posttreatment recurrence (8).

In a review by Bricelj et al., recurrence was observed in 26.9% of patients after treatment (28). In our patients, the median follow-up period was 26 months and no recurrence occurred, regardless the type of surgery, except for one patient (12.5%), approximately 32 months from surgery. This woman had discontinued medical therapy for endometriosis and presented with a recurrence of marginal pneumothorax, which did not require treatment with pleural drainage but was simply and successfully treated with bed rest.

Two out of eight patients underwent gynecologic surgery (hysteroannessiectomy) for recurrence of pelvic endometriosis due to unresponsiveness to medical treatment. In another case, the patient had estrogen–progestin therapy for 6 months only, according to the gynecological specialist indication, presenting a relapse-free period, in the absence of medical therapy for 16 months.

The combination of hormonal treatment with surgical approach turns out to be crucial for the diagnostic framing of the patient, the proper gynecologic specialist treatment of the patient, and lowering the risk of disease recurrence.

To the best of our knowledge, our study is the first to evaluate the specific role of VATS in CP not only to obtain visual diagnosis of endometriotic lesions but also to select the most appropriate surgical treatment and particularly to provide pathological specimens for definitive diagnosis of thoracic endometriosis.

One limitation of our study is the low number of cases, mainly related to the rarity of CP. However, due to the lack of guidelines in diagnosis and treatment of TES and CP, multicenter studies are recommended in order to define guidelines shared by thoracic surgeons and gynecologists, for a correct and optimal diagnostic and therapeutic management of these patients.

## 5. Conclusions

In the management of patients with CP, VATS should be recommended not only to obtain an explorative diagnosis of ectopic endometrial implants or diaphragmatic fenestrations but also to allow the most appropriate surgical treatment and obtain pathological specimens for confirmation and definitive diagnosis of endometriosis. Gynecological consultation is recommended and medical therapy to achieve ovarian rest is mandatory in the postoperative period and should not be discontinued, in order to reduce the risk of recurrence.

## Data Availability

The raw data supporting the conclusions of this article will be made available by the authors, without undue reservation.
